# The Discovery of α-Adrenoceptor Antagonists as a Potential New Treatment Option for Uveal Melanoma

**DOI:** 10.3390/biom15101436

**Published:** 2025-10-10

**Authors:** Yilan He, Hongtao Liu, Ulrike Hendgen-Cotta, Tienush Rassaf, Nikolaos E. Bechrakis, Utta Berchner-Pfannschmidt

**Affiliations:** 1 Department of Ophthalmology, University Hospital Essen, University Duisburg-Essen, Hufelandstr. 55, 45147 Essen, Germany or yilan.he.doctor@gmail.com (Y.H.); hongtao.liu.doctor@outlook.com (H.L.); nikolaos.bechrakis@uk-essen.de (N.E.B.); 2 Nanchong Hospital, Beijing Anzhen Hospital, Capital Medical University, Renmin South Road No. 97, Nanchong 637000, China; 3 Ophthalmology Center, Zhoushan Hospital, Wenzhou Medical University, Dingshen Road No. 739, Zhoushan 316021, China; 4 Department of Cardiology and Vascular Medicine, West German Heart and Vascular Center, University Hospital Essen, University Duisburg-Essen, Hufelandstr. 55, 45147 Essen, Germany; ulrike.hendgen-cotta@uk-essen.de (U.H.-C.); tienush.rassaf@uk-essen.de (T.R.)

**Keywords:** uveal melanoma, metastasis, adrenergic receptor, α-blockers, prazosin, doxazosin, α-agonist, phenylephrine, tumor spheroids, drug screening

## Abstract

Uveal melanoma (UM) is the most common primary intraocular tumor in adults. Because of its high potential for spreading and its limited response to treatment, UM remains a clinical challenge. Previous studies suggest that clinical adrenergic receptor (AR) antagonists might be effective in the treatment of UM. This study reports the antitumor activity of α-blocker in UM spheroids generated from α_1A_- and α_2A_-AR-positive UM cell lines. These cell lines were derived from primary tumors or hepatic metastases and differed in their genetic risk status for metastasis. Drug screening with UM spheroids revealed that prazosin and doxazosin dose-dependently reduced viability, whereas terazosin, alfuzosin, silodosin, tamsulosin, and phenoxybenzamine were found to be inefficient. Prazosin induced apoptosis, resulting in the disintegration of UM spheroid morphology and growth inhibition. Additionally, prazosin prevented UM spheroid cell outgrowth and long-term survival, indicating potential for tumor control. Like the selective α_1A_-AR antagonist RS17053, prazosin inhibited the formation and growth of UM spheroids stimulated by the α_1-_agonist phenylephrine. This suggests a tumor-preventive effect through the blockade of α_1A_-AR. The present study highlights the responses of UM spheroids to α-AR antagonists and demonstrates that prazosin, doxazosin, or RS17053 may be a treatment option for preventing UM tumor recurrence or metastasis.

## 1. Introduction

Uveal melanoma (UM) is the most common primary intraocular malignancy in adults, representing approximately 85% of all ocular melanomas [1]. Despite its rarity, with an annual incidence of about four to five cases per million population, UM exhibits a highly aggressive clinical behavior. Nearly half of all patients develop distant metastases, predominantly in the liver, but other metastatic sites include the lungs, brain, skin, bone, and lymph nodes, often leading to a dismal prognosis with median survival of less than one year following detection of metastasis [1,2]. Although current treatment strategies such as brachytherapy and enucleation offer effective local control of the primary tumor, they fail to prevent metastatic spread or address systemic disease [3].

Unlike cutaneous melanoma, UM is largely resistant to immune checkpoint inhibitors. The poor responsiveness to immunotherapy has been attributed to a combination of biological factors, including a low tumor mutational burden, limited antigen presentation, and an immunosuppressive tumor microenvironment with reduced lymphocyte infiltration and minimal inflammatory signaling [4]. These immunological features present a challenge for developing effective systemic therapies, underscoring the need for alternative treatment strategies beyond current immune-based approaches.

Though the detailed molecular pathways underlying the development of metastatic UM remain incompletely understood, specific gene mutations have been identified as prognostic indicators. SF3B1 and BAP1 mutations correlate with late and early metastasis, respectively, whereas EIF1AX mutations are associated with low metastatic risk and favorable survival outcomes [4,5]. Among these, BAP1 mutations are particularly notable. The BAP1 gene, located on chromosome 3, encodes BRCA1-associated protein 1 and functions as a tumor suppressor [6]. Studies have shown that up to 87% of UM tumors with monosomy 3 harbor BAP1 alterations, while others display decreased BAP1 mRNA expression, further linking its loss to metastatic progression and poor prognosis [4,5,7]. In contrast, the pathogenic mutations in GNAQ and GNA11 (encoding for guanine nucleotide-binding protein alpha subunits Gq and G_11_) are not associated with a higher risk of metastasis development [8].

Adrenergic signaling has recently emerged as a critical regulatory mechanism in cancer progression. Mediated by catecholamines such as epinephrine and norepinephrine, adrenergic pathways modulate a wide range of tumor-promoting processes through G protein-coupled β-adrenergic receptors (β-ARs). In various malignancies, including breast, ovarian, and cutaneous melanoma, β-AR activation enhances proliferation, invasion, angiogenesis, and resistance to therapy [9]. Switzer et al. (2024) provided direct evidence supporting the role of β-ARs in melanoma progression, showing that β-adrenergic blockade may suppress these oncogenic mechanisms and improve tumor control [10]. ß-adrenoceptors were also suggested as therapeutic targets for ocular tumors [11]. Our own studies have shown that among the various β-blockers tested, carvedilol and nebivolol have the most effective long-term antitumor effects in UM [12,13].

While β-ARs have been the primary focus of adrenergic-targeted cancer research, recent studies suggest that alpha-adrenergic receptors (α-ARs), particularly the α_1_ subtype, may also play a role in modulating tumor biology. α-ARs are expressed not only in smooth muscle but also in endothelial and immune cells, where they regulate vascular tone, leukocyte recruitment, and cytokine secretion [14]. In addition to direct cytotoxicity, α_1_-AR antagonists have been reported to induce apoptosis through caspase activation and modulation of mitochondrial membrane potential, independent of classical adrenergic receptor pathways [15]. Importantly, several FDA-approved α_1_-AR antagonists, such as prazosin and doxazosin, and naftopidil, have demonstrated antitumor properties in preclinical studies. These agents have been shown to induce apoptosis, inhibit angiogenesis, and modulate immune activation in several cancer models [16].

Broso et al. (2023) demonstrated that α_1_-AR blockade with doxazosin sensitized neuroblastoma cells to differentiation therapy, leading to reduced tumor growth [17]. Similarly, Gotoh et al. (2012) highlighted the potential of repurposing α_1_-AR antagonists as cancer therapeutics due to their favorable safety profile and pleiotropic antitumor effects [18]. Despite growing evidence supporting the antitumor effects of α_1_-AR blockade in multiple cancer types, its role in UM has not yet been systematically explored. Given the aggressive nature of UM and the limitations of current systemic therapies, investigating the potential therapeutic value of α_1_-AR antagonists in this context is both timely and necessary. We assess the expression of α-ARs in UM cell lines and examine the impact of α-blockers on cell viability, apoptosis, long-term survival, and formation ability in three-dimensional (3D) tumor spheroid models of UM (UM spheroids). Three-dimensional tumor spheroids offer a promising approach for enhancing the approval rate for cancer drugs, as they bridge the gap between in vitro tests, animal models, and clinical trials [19]. Our findings aim to determine whether inhibition of α-AR signaling could serve as a viable therapeutic approach in UM and provide a rationale for further translational and clinical investigation.

## 2. Materials and Methods

### 2.1. Characteristics of Uveal Melanoma Cell Lines and Cell Culture

For representative results in this study, a total of seven different UM cell lines with different genetic profiles and cell characteristics such as morphology and doubling time, which may influence spheroid growth and response to drugs, were included in this study (Table 1). Cell lines 92.1 (CVCL_8607), UPMD1 (CVCL_C297), UPMD2 (CVCL_C258), UPMM2 (CVCL_C294), and UPMM3 (CVCL_C295) originated from primary untreated tumors and were kindly provided by Dr. M. Zeschnigk (Institute of Human Genetics, University Hospital Essen, Germany). UPMD1, UPMD2, UPMM2, and UPMM3 were provided in low passages [20]. The UM cell line Mel270 (CVCL_C302) originated from a primary recurrence after radiation therapy and was kindly provided by Dr. K. Griewank (Department of Dermatology, University Hospital Essen, Germany). Cell line MM28 (CVCL_4D15) originated from a liver metastasis and was obtained from the American Type Culture Collection (ATCC, Manassas, VA, USA). Short tandem repeat profiling was performed for all cell lines, according to published data (Table 1).

Cell lines 92.1 and Mel270 were maintained in RPMI 1640 medium supplemented with L-Glutamine (GIBCO, Fisher Scientific, Thermo Fisher Scientific Inc., Waltham, MA, USA), and UPMD1, UPMD2, UPMM2, and UPMM3 were kept in Hams/F12 medium supplemented with L-Glutamine and 1.176 g/L NaHCO_3_ (PAN-Biotech GmbH, Aidenbach, Deutschland). Cell line MM28 was maintained in RPMI 1640 medium supplemented with 4.5 g/L D-Glucose, 2.383 g/L HEPES buffer, 1.5 g/L sodium bicarbonate, and 110 mg/L sodiumpyruvate (GIBCO, Fisher Scientific, Thermo Fisher Scientific Inc., Waltham, MA, USA). Each medium was supplemented with 10% fetal calf serum and 1% penicillin-streptomycin (5000 U/mL). The cell lines were kept in a humidified incubator (37 °C, 5% CO_2_) and the medium was refreshed two times a week.

### 2.2. Generation of 3D Tumor Spheroids

Tumor spheroids were generated in round-bottom 96-well ultra-low-attachment plates, with 5 × 10^3^ living cells in each well, as described earlier [12,13,29]. The spheroid cultures were maintained in a humidified incubator (37 °C, 5% CO_2_) for the indicated period.

### 2.3. Drug Treatment

The α-blockers prazosin-hydrochloride (CAS No.: 19237-84-4), tamsulosin-hydrochloride (CAS No.: 106463-17-6), phenoxybenzamine-hydrochloride (CAS No.: 63-92-3), and the α1-adrenoceptor agonist (R)-(-)-phenylephrin-hydrochloride (CAS No.: 61-76-7) were purchased from Sigma Aldrich (St. Louis, MO, USA/Chemie GmbH, Steinheim, Germany). The α-blockers doxazosin mesylate (CAS No.: 77883-43-3), alfuzosin-hydrochloride (CAS No.: 81403-68-1), terazosin-hydrochloride (CAS No.: 63074-08-8), silodosin (CAS No.: 160970-54-7), and RS17053-hydrochloride (CAS No.: 169505-93-5) were purchased from Tocris Bioscience (Bristol, UK). Each blocker was prepared in a stock solution of 50 mM in dimethyl sulfoxide (DMSO). The UM spheroids were incubated in the respective medium supplemented with various concentrations of drugs for the indicated period. Control spheroids (0 µM drug) received DMSO at the same concentration as present in the highest concentration of the drug in the respective assays.

### 2.4. Determination of Spheroid Growth

Imaging of spheroid cultures (*n* = 3–4 spheroids each condition) was conducted using a Zeiss Primovert bright-field microscope at 4× magnification equipped with a Zeiss Axiocam 105 (Oberkochen, Germany) and Zeiss ZENcore software 3.12 (Oberkochen, Germany). Images were analyzed by using image processing software Fiji ImageJ 1.53c (MPI-CBG, Dresden, Germany). Changes in spheroid growth were reflected by the size and compactness of the spheroids. In order to determine the size and compactness of the spheroids, the cross-sectional area and the optical density of the spheroids were measured. The integrated density (IntDensity) was calculated by cross-sectional area x optical density and is given in arbitrary units (AU).

### 2.5. Spheroid Viability Assay

The CellTiter-Glo 3D Cell Viability Assay (Promega GmbH, Walldorf, Germany) was used to determine the viability of UM spheroids (*n* = 6–8 per condition) by measuring the ATP content of the examined spheroids, as described before [12,13]. The resulting ATP luminescence (relative light units) was recorded with a FluostarOmega reader (BMG LABTECH, Ortenberg, Germany). The ATP luminescence of treated spheroids was normalized to the ATP luminescence of control spheroids after background subtraction and is given in arbitrary units (AU).

### 2.6. Spheroid Apoptosis Assay

The Caspase-Glo 3/7 Assay (Promega GmbH, Walldorf, Germany) was used to determine spheroid cell apoptosis (*n* = 6–8 spheroids each condition) by detecting caspase 3/7 activity in spheroids, as described before [12,13]. The resulting caspase 3/7 luminescence (relative light units) was recorded using a reader FluostarOmega (BMG LABTECH, Ortenberg, Germany). The caspase 3/7 luminescence of treated spheroids was normalized to the caspase 3/7 luminescence of control spheroids and is given in arbitrary units (AU).

### 2.7. Spheroid Outgrowth Assay

UM spheroid cultures (*n* = 8 each condition) were individually transferred to flat-bottom 24-well plate dishes (Cellstar, Greiner Bio-One GmbH, Frickenhausen, Germany) after four days of treatment. Spheroid cells were allowed to attach to the uncoated flat plastic bottom to enable cell outgrowth and repopulation. Cells were cultured until the control cells were 90% confluent. Medium was refreshed at least once per week. Finally, adherent cells were fixed and stained with crystal violet (CV), as described before [12,13]. Absorbance of CV (relative light units) was measured at OD 540 nm using a reader ClarioStar Plus (BMG LABTECH, Ortenberg, Germany). The CV absorbance of treated spheroids was normalized to the CV absorbance of control cultures and is given in arbitrary units (AU).

### 2.8. Spheroid Formation Assay

Spheroids were generated in round-bottom 96-well ultra-low-attachment plates (Corning, NY, USA) by seeding 5 × 10^3^ living cells in 100 µL of the cell culture medium per well. The cell culture medium contained 2% FCS and various concentrations of phenylephrine and prazosin or RS17053 as indicated. Control spheroids (0 µM drug) received DMSO at the same concentration as present in the highest concentration of the drug in the respective assays. The spheroid cultures were maintained in a humidified incubator (37 °C, 5% CO_2_) for 7 days.

### 2.9. Immunofluorescence Microscopy

For the detection of α-ARs, 10^5^ cells were grown on coverslips placed in 24-well plates. Cells were fixed and immunostained as described before [12] using the primary antibodies rabbit anti-ADRA1A (RRID: AB 10643238, 1:100, Proteintech, Planegg-Martinsried, Germany) or rabbit anti-ADRA2A (RRID: AB_2636822, 1:50, Proteintech, Planegg-Martinsried, Germany) and the secondary antibody Alexa fluor 594 goat anti-rabbit antibody (RRID: AB_2534095, 1:400, Invitrogen, Thermo Fischer Scientific Inc., Waltham, MA, USA). Specimens were embedded with ProLong Gold Antifade Mountant (Invitrogen, Thermo Fischer Scientific Inc., Waltham, MA, USA). Immune-stained cells were imaged using an Olympus BX51 epifluorescence microscope at 40× magnification and images were recorded with Olympus DP70 1.5 Megapixel color ccd camera.

### 2.10. Statistical Analysis

GraphPad Prism (GraphPad Prism 8.4.3 software, GraphPad Software Inc., San Diego, CA, USA) was used for statistical analyses of the data. Data were analyzed by one-way ANOVA or two-way ANOVA and Tukey’s multiple comparisons test and considered statistically significant at a value of *p* < 0.05. The significance levels are as follows: * *p* < 0.05, ** *p* < 0.01, *** *p* < 0.001, and **** *p* < 0.0001.

## 3. Results

### 3.1. An α-Blocker Screen in UM Spheroids Reveals Prazosin as a Top Hit

**Figure 1:**
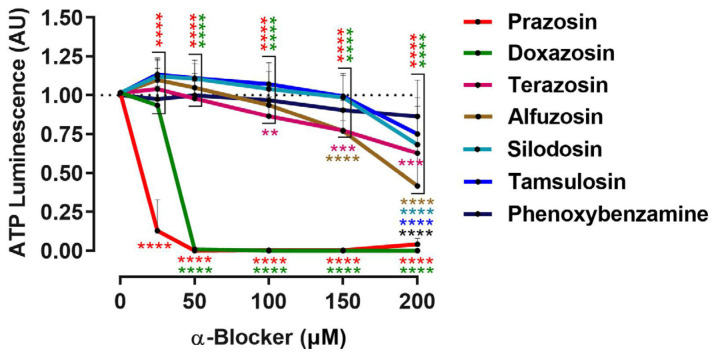
UM spheroid-based drug screening for α-blockers with potential antitumor activity. Concentration–response curves of UM spheroids generated from a Mel270 cell line. Spheroid viability was determined using an ATP luminescence assay after treatment for 7 days. The means +/− SD of at least three independent experiments for each α-blocker with *n* = 6–8 spheroids at each concentration is shown. Statistical analysis by two-way ANOVA and Tukey’s multiple comparisons test; the significance levels of the treated spheroids compared to the control spheroids (0 µM α-blocker) are colored and displayed under the respective drug response curves. The significance levels of the spheroids treated with prazosin or doxazosin compared to all other blockers are indicated above the response curves. Significance levels are indicated as follows: ** *p*< 0.01, *** *p* < 0.001, and **** *p* < 0.0001.

To identify α-blockers with antitumor activity, we treated UM spheroids once with various clinical available α-blockers in a concentration range of 0–200 µM and analyzed spheroid viability after 7 days (Figure 1).

For this purpose, we used our Mel270 screening model cell line to generate large and uniform tumor spheroids, as already established in previous studies [12,13,29]. The Mel270 cell line was obtained from a recurrent tumor after radiation therapy (Table 1) and has been proven relatively radiation-resistant in a former study [29]. Additionally, tumor nodules grown from Mel270 cells showed a high spontaneous metastatic capacity in a chicken embryo model, indicating a highly aggressive cell line [30].

The α_1_-blockers prazosin at ≥25 µM and doxazosin at ≥50 µM inhibited Mel270 spheroid viability, while all other blockers reduced viability to a much lesser extent in the concentration range of 100 to 200 µM (Figure 1). Herein, the α_1_-blockers terazosin at ≥100 µM, alfuzosin at ≥150 µM, and silodosin or tamsulosin at 200 µM significantly reduced spheroid viability. Among all the blockers tested, the α_1_, α_2_ blocker phenoxybenzamine reduced the viability of the spheroids the least at a concentration of 200 µM (Figure 1). Prazosin proved the most effective compared to the α-blockers tested (Figure 1, *p* > 0.0001).

### 3.2. Prazosin and Doxazosin Exert AntiTumor Responses in Primary and Metastatic UM

**Figure 2:**
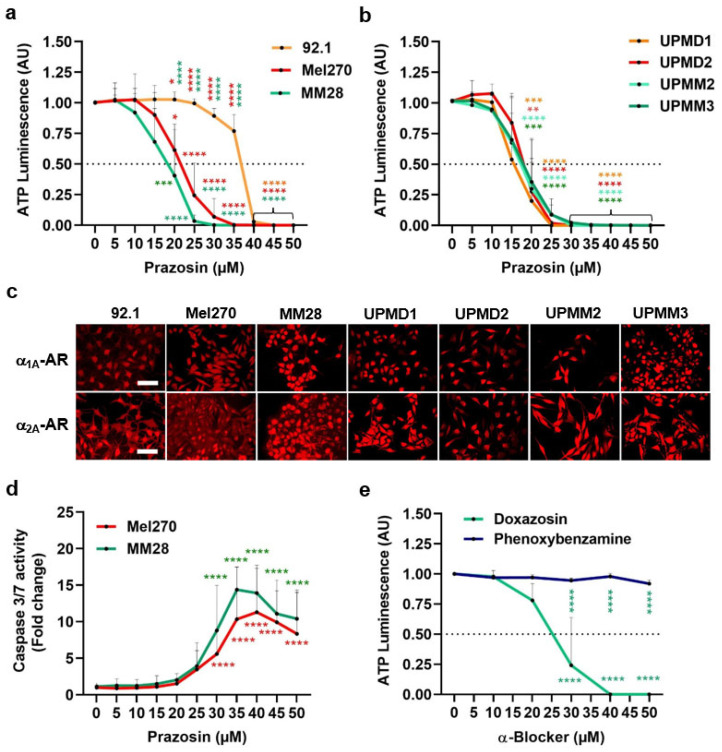
The α-blockers prazosin and doxazosin exert antitumor activity in primary and metastatic UM spheroids with distinct genetic high-risk status. (**a**,**b**) Spheroid viability was determined using an ATP luminescence assay after 7 days of incubation with prazosin. (**a**) Spheroids were generated from cell lines of primary (92.1, Mel270) or liver metastasis origin (MM28). (**b**) Spheroids were generated from primary cell lines with either disomy-3 (UPMD1, UPMD2) or monosomy-3 and BAP1-deficient cell lines (UPMM2, UPMM3). (**c**) Cell lines were immune-stained for α_1A_- or α_2A_-adrenoceptors; representative microscopic images are shown and immunofluorescence is displayed in red, with the scale bar representing 100 µm. (**d**) Mel270 and MM28-derived spheroids were treated with prazosin at indicated concentrations for 36 h, and caspase3/7 activity was measured to determine apoptosis. (**e**) MM28-derived spheroids were treated with doxazosin or phenoxybenzamine at indicated concentrations for 7 days and spheroid viability was determined using an ATP luminescence assay. (**a**,**b**,**d**,**e**) Shown are the means +/− SD of at least three independent experiments for each spheroid type with *n* = 6–8 spheroids per condition. Statistical analysis was conducted using two-way ANOVA and Tukey’s multiple comparisons test. The significance levels of treated spheroids relative to control spheroids (0 µM α-blocker) are shown in the respective colors. Significance levels are indicated at * *p* < 0.05, ** *p* < 0.01, *** *p* < 0.001, and **** *p* < 0.0001. The significance level of α-blocker-treated spheroids relative to other spheroid types is indicated vertically in the respective color at * *p* < 0.05 and **** *p* < 0.0001.

Since prazosin reduced Mel270 spheroid viability most efficiently, we next investigated the antitumor activity of prazosin in seven genetically distinct GNAQ/11-mutant UM spheroid types (Figure 2).

We hence generated UM spheroids from a panel of well-established patient-derived cell lines of different origins (Table 1). These cell lines differ genetically, as well as in terms of cell morphology and proliferation rate, which may account for individual drug responses (Table 1). Spheroids grown from another primary UM cell line 92.1 and a UM liver metastasis-derived cell line MM28 were compared with Mel270 spheroids (Figure 2a). The viability of these spheroid types was diminished by prazosin treatment in a concentration range of 20–40 µM (Figure 2a). The viability of MM28 spheroids or Mel270 spheroids was diminished in a concentration range of 20–30 µM prazosin, while primary 92.1 spheroids needed a higher dose when compared to both spheroid types (Figure 2a, *p* < 0.001).

As the metastatic MM28 cell line also harbors chromosome-3 aberration and BAP1 deficiency, we next compared primary cell line-derived spheroids with either disomy-3 genetic (UPMD1, UPMD2) or monosomy-3/BAP1 deficiency genetic high-risk status for metastasis (UPMM2, UPMM3) (Figure 2b). The viability of these spheroid types was decreased by prazosin treatment in a concentration range of 20–30 µM independently of genetic status (Figure 2b). Among the primary derived spheroid types with disomy-3 genetic, the 92.1 spheroids were found to be less responsive to prazosin treatment. 92.1 spheroid viability was sharply reduced in response to ≥35 µM and blocked with 40 µM prazosin, whereas the viability of the Mel270 spheroids was already blocked with 30–35 µM prazosin (Figure 2a). The liver metastasis-derived MM28 spheroids and the primary derived UPMD1, UPMD2, UPMM2, and UPMM3 spheroids were the most responsive once, as viability was significantly reduced with ≥20 µM and blocked with 25–30 µM prazosin (Figure 2a,b).

Since clinical α-blocker affinities are high to AR subtypes α_1A_ or α_2A_ [31,32], we determined the presence of these α-adrenoceptors on cell types by immunofluorescence (Figure 2c). All the cell lines were stained positive for both α_1A_- and the α_2A_-adrenoceptors (Figure 2c).

We next tested the ability of prazosin to induce apoptotic cell death in primary derived Mel270 compared to metastatic MM28 spheroids. Using caspase-3/7 activity as a measure of apoptosis, we observed significant induction of apoptosis at ≥30 µM in both spheroid types, Mel270 and MM28 (Figure 2d). The maximum caspase activity was measured at a prazosin concentration of 35–40 µM in both spheroid types (Figure 2d), aligning with the viability results (Figure 2a). Furthermore, similar to the Mel270 spheroids (Figure 1), the MM28 spheroids turned out responsive to α_1-_blocker doxazosin in a comparable concentration range at 25–50 µM and non-responsive to nonselective α_1_,α_2_-blocker phenoxybenzamine (Figure 2e). The data indicate that the clinically selective α_1_-blockers prazosin and doxazosin exert antitumor activity for primary and metastatic UM with genetic high-risk status monosomy-3/BAP1 deficiency.

### 3.3. Prazosin Impacts UM Tumor Growth and Structure in Spheroid Models

**Figure 3:**
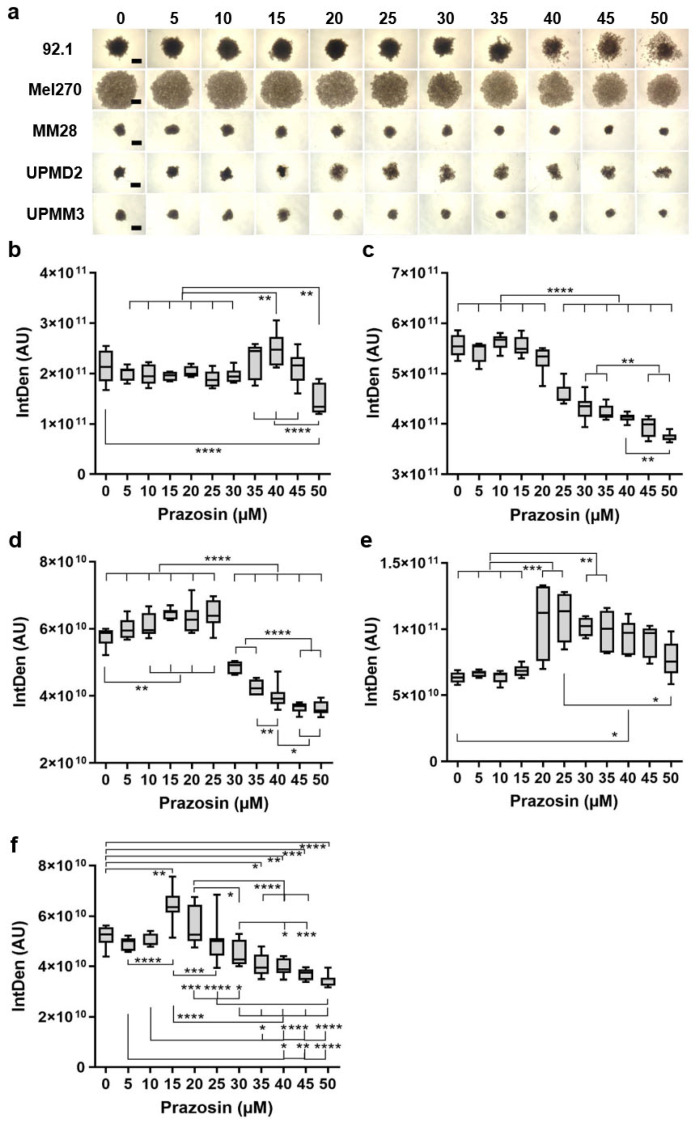
Morphological changes of UM spheroid types by prazosin treatment. 92.1, Mel270, MM28, UPMD2, UPMM2, or UPMM3-derived spheroids were incubated with prazosin at the indicated concentrations for 7 days. The spheroids were microscopically imaged. (**a**) Representative microscopic images of the spheroids treated with 0–50 µM prazosin are shown; scale bars indicate 500 µm. Image analysis of prazosin-treated spheroids grown from cell lines (**b**) 92.1, (**c**) Mel270, (**d**) MM28, (**e**) UPMD2, and (**f**) UPMM3. (**b**–**e**) Changes in spheroid size and density were combined and expressed as the integrated density (IntDen, AU). Box plots with min to max whiskers and a median of at least three independent experiments for each spheroid type with *n* = 3–5 spheroids for each concentration are shown. One-way ANOVA and Tukey’s multiple comparisons test were used for statistical analyses, and significance levels are indicated at * *p* < 0.05, ** *p* < 0.01, *** *p* < 0.001, and **** *p* < 0.0001.

We next assessed the effects of prazosin on the growth and morphology of several spheroid types by microscopy (Figure 3).

In agreement with our previous studies [12,13,29], the various cell lines yielded spheroid types that differed in size and compactness (Figure 3a). The 92.1 and Mel270 cell lines yielded spheroids of large size, whereas the MM28 cell line yielded spheroids of small size, similar to UPMD2 and UPMM3 spheroids (Figure 3a). The spheroid size correlated to the doubling time of the cell lines and appeared independently of the cytogenetic status of chromosome 3 or BAP1 (Table 1). Treatment with prazosin resulted in concentration-dependent changes in spheroid size and compactness to different degrees in the spheroid types (Figure 3a), which we analyzed in a combined manner given as intensified density (IntDen) (Figure 3b–f).

Prazosin concentrations ≥ 40 µM increased 92.1 spheroid IntDen, while at 50 µM the IntDen was statistically significant reduced compared to the control spheroids (Figure 3b). In agreement with the changes in IntDen, the microscopic images of 92.1 showed increasing disintegration of the spheroids with ≥40 µM prazosin (Figure 3a). Cell line Mel270-derived spheroids, however, only required prazosin concentrations of ≥25 for the reduction in IntDen, reflecting a concentration-dependent decrease in spheroid size and compactness (Figure 3a,c). In contrast, at a concentration of ≥10 µM prazosin, the IntDen of metastatic MM28 spheroids statistically significant increased and decreased again at ≥30 µM (Figure 3d). Microscopic observation of MM28 spheroids at 10–25 µM exhibited a blurred spheroid shape, indicating disintegration of the outer cell layers, while prazosin at ≥30 µM decreased the spheroid size (Figure 3a).

The IntDen of UPMD2 statistically significant increased at ≥20–25 µM and decreased again at 50 µM when compared to 25 µM (Figure 3e). In line with the changes in IntDen, the microscopic images of UPMD2 showed increasing disintegration of the spheroids with ≥20 µM prazosin (Figure 3a). Likewise, the changes in UPMM3 spheroid morphology led to a statistically significant increase in IntDen at ≥15 µM and a decrease with ≥35 µM prazosin when compared to control spheroids (Figure 3a,f).

The observed changes in IntDen values of the spheroids were dose-dependent and correlated predominantly with decreasing viability (Figure 2). A comparison of the different spheroid responses showed that the IntDen of the prazosin-treated spheroids was significantly reduced compared to untreated controls at 50 µM in 92.1, at ≥35 µM in Mel270, at ≥30 µM in MM28, and at ≥35 µM in UPMM3. This reduction in the IntDen of the spheroids was accompanied by a sharp decline (to below ≤25%) in viability (Figure 2a,b). However, the IntDen value of the treated UPMD2 spheroids was not reduced compared to the untreated control spheroids, but increased due to the disintegration of the spheroids at 20–35 µM, while viability decreased significantly to below ≤25%. (Figure 2b). Similarly, the IntDen values of spheroids 92.1, MM28, and UPMM3 were elevated at lower prazosin concentrations (at 40 µM in 92.1, at 10–25 µM in MM28, and at 15 µM in UPMM3). However, the increase in IntDen values of MM28 at 10–15 µM and UPMM3 at 15 µM was not accompanied by a significant reduction in viability (Figure 2a,b), suggesting that the effects of prazosin at such low doses were limited to the outer layers of the spheroids. Taken together, the morphology of all spheroid types was affected, suggesting inhibition of tumor spheroid growth and disintegration of 3D structure by prazosin.

### 3.4. Prazosin Enables Tumor Control Potential for UM

**Figure 4:**
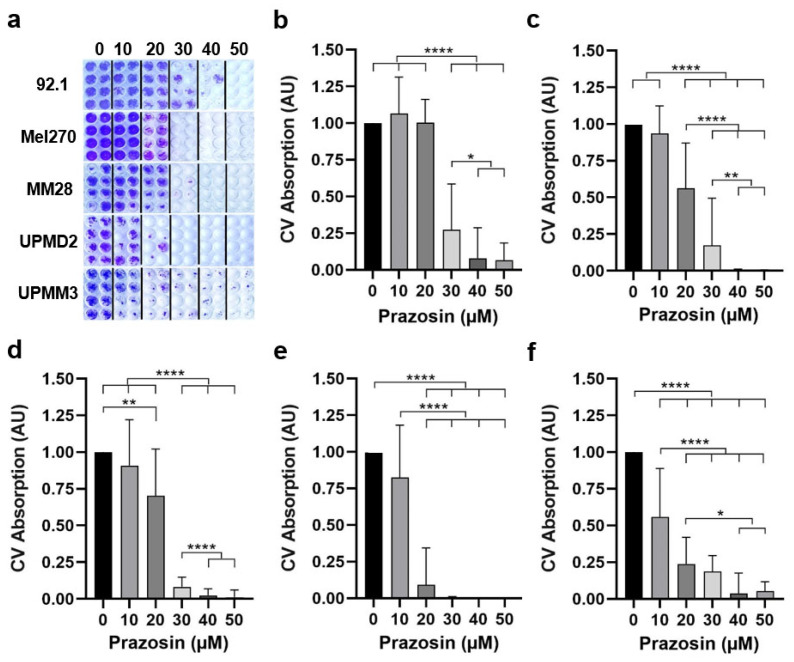
Pretreatment of UM spheroids with prazosin showed potential for tumor control in UM. Spheroids from various cell lines were treated with prazosin at the indicated concentrations for 4 days. Spheroid cultures were individually transferred to flat-bottom wells to allow for outgrowth and repopulation of cells. Surviving cells were stained with crystal violet (CV). (**a**) Representative images of CV-stained cell cultures (*n* = 8) for each concentration are shown. (**b**–**e**) Absorption of CV-stained cell cultures of (**b**) 92.1, (**c**) Mel270, (**d**) MM28, (**e**) UPMD2, and (**f**) UPMM3. Data are the means ± SD of three independent experiments with *n* = 8 spheroids for each concentration. Statistical analysis was performed with one-way ANOVA and Tukey’s multiple comparisons test and significance levels are indicated at * *p* < 0.05, ** *p* < 0.01, and **** *p* < 0.0001.

In order to determine the tumor control potential of prazosin in UM, we investigated long-term cell survival of spheroid types upon pretreatment with prazosin for four days. Thereafter, each pretreated spheroid culture was individually seeded in a flat-bottom well to allow for cell outgrowth and repopulation until control cells were confluent (Figure 4).

Cell survival and repopulation of spheroid types were concentration-dependently reduced to different degrees by prazosin (Figure 4a). A statistically significant reduction in repopulation was achieved at prazosin concentrations of ≥30 µM with 92.1 spheroids (Figure 4b), at ≥20 µM with Mel270, MM28, and UPMD2 spheroids (Figure 4c–e), and at ≥10 µM with UPMM3 spheroids (Figure 4f). However, a complete blockade of tumor cell survival (CV values ≤ 0.01) following treatment with prazosin was observed at ≥30 µM with UPMD2 (Figure 4e), at ≥40 µM with Mel270 (Figure 4c), and at 50 µM with MM28 spheroids (Figure 4d). Microscopic observation of these cell cultures revealed that no tumor cells remained (Figure 4a). In contrast, some residual 92.1 and UPMM3 cells remained in a few cultures at 50 µM (Figure 4a,b,f, CV values ≥ 0.01 and ≤0.1). The data suggest that a prazosin concentration of ≥30 µM allowed for tumor cell death and long-term tumor control dependent on spheroid type.

### 3.5. Prazosin Prevents Tumor Formation in UM

**Figure 5:**
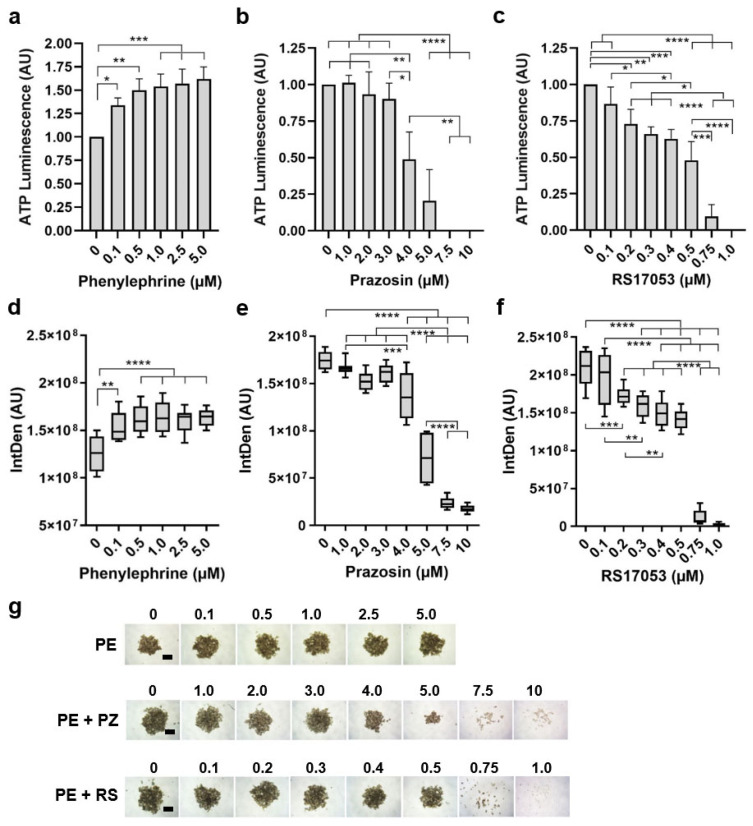
Spheroid formation ability of UM cells in response to α_1_-adrenergic stimulation and prevention by prazosin or RS17053. (**a**–**c**) Changes in UM spheroid viability and (**d**–**g**) morphology after spheroid formation from Mel270 cells in the presence of (**a**,**b**,**g**) various concentrations of the α_1_-agonist phenylephrine or (**b**,**e**,**g**), in the presence of 2.5 µM phenylephrine and various concentrations of prazosin, or (**c**,**f**,**g**) in the presence of 2.5 µM phenylephrine and various concentrations of RS17053. (**a**–**c**) Spheroid viability was determined using an ATP luminescence assay. The means +/− SD of three independent experiments with *n* = 6–8 spheroids for each concentration are shown. (**d**–**f**) Changes in spheroid size and density are expressed as integrated density (IntDen, AU). Box plots with min to max whiskers and a median of three independent experiments with *n* = 3–5 spheroids for each concentration are shown. (**a**–**f**) Statistical analysis was performed using one-way ANOVA and Tukey’s multiple comparisons test; the significance levels of the treated spheroids in relation to the control spheroids are indicated at * *p* < 0.05, ** *p* < 0.01, *** *p* < 0.001, and **** *p* < 0.0001. (**g**) Representative microscopic images of resultant spheroids under each condition (**d**–**f**) and at the respective concentrations (µM) are shown for phenylephrine (PE) and prazosin (PZ), RS17052 (RS), and the scale bars indicate 500 µm.

In order to determine the ability of prazosin to prevent tumor growth under conditions of specific α_1_-receptor adrenergic stimulation, the selective α_1_-agonist phenylephrine was added to the Mel270 cultures during spheroid formation for 7 days (Figure 5).

During spheroid formation, phenylephrine at ≥0.1 µM concentration-dependently increased spheroid viability and spheroid growth (Figure 5a,c,g). Next, we added various concentrations of prazosin to 2.5 µM phenylephrine during spheroid formation (Figure 5b,e,g). The addition of prazosin at ≥4.0 µM statistically significantly reduced spheroid viability and growth in the presence of 2.5 µM phenylephrine. Prazosin at concentrations ≥7.5 µM completely blocked spheroid viability and formation (Figure 5b,e,g). Moreover, we added the α_1A_-AR selective antagonist RS17053 to 2.5 µM phenylephrine during spheroid formation (Figure 5c,f,g). RS17053 decreased spheroid viability and growth at concentrations ≥0.2 µM and completely blocked spheroid viability and formation at ≥0.75 µM (Figure 5c,f,g). In summary, prazosin and RS17053 prevented phenylephrine-stimulated spheroid formation and growth, with a potency order of RS17053 > prazosin.

## 4. Discussion

In this study, we investigated the antitumor potential of several α-AR antagonists in UM spheroids, including prazosin, doxazosin, terazosin, alfuzosin, silodosin, tamsulosin, and phenoxybenzamine. These agents were selected based on their well-defined pharmacological characteristics and established clinical applications, which allowed us to explore the potential therapeutic relevance of α-AR modulation in UM.

Among the compounds tested, the α_1_-AR selective blockers prazosin and doxazosin demonstrated the most pronounced inhibitory effect on tumor cell growth in our experimental models, while the α_1,_α_2_-blocker phenoxybenzamine showed almost no effect (Figure 1 and Figure 2), implying that the α_1_-AR subtype might be a target for UM treatment. In line with this, our results show that prazosin and doxazosin reduced viability and proliferation in 3D cultures (Figure 1 and Figure 2), inducing marked cytotoxic effects across multiple assays, including the spheroid outgrowth assay or formation assay (Figure 3, Figure 4 and Figure 5). Notably, early administration of prazosin at the time of spheroid initiation produced greater growth inhibition compared to the treatment of established spheroids (Figure 2, Figure 3 and Figure 5). Two possible mechanisms may account for this effect. First, early drug exposure occurs before compact spheroid architecture generates diffusion and retention barriers, enabling more uniform drug penetration and sufficient concentrations at lower doses. Second, administration during the early assembly phase may disrupt initial signaling events that facilitate cellular adaptation and persistence within the 3D structure, leading to a stronger growth suppressive effect. This interpretation is supported by previous work demonstrating that spheroid age and size can impact drug diffusion and thus cytotoxicity [33]. Additionally, α_1_-AR antagonists have been shown to disrupt focal adhesion and survival signaling, induce anoikis, and suppress invasion in other cancer models [34,35]. These results highlight that both the timing of drug administration and the structural characteristics of the tumor can significantly influence treatment response.

Our findings also revealed distinct differences in drug efficacy between various UM spheroid types (Figure 2, Figure 3 and Figure 4). MM28 spheroids, derived from a hepatic metastasis, showed a reduction in viability at prazosin concentrations as low as 20–30 μM, similar to primary tumor-derived Mel270 spheroids. In contrast, spheroids from the primary tumor cell line 92.1 required substantially higher concentrations to achieve comparable effects. Spheroids derived from the hepatic metastasis cell line MM28, as well as from the primary tumor cell lines UPMD1, UPMD2, UPMM2, and UPMM3, exhibited the highest susceptibility, with viability significantly reduced even below 20 μM and complete growth arrest at 25–30 μM (Figure 2). However, regardless of genetic status or other known cell characteristics (Table 1), or spheroid morphology (Figure 3), higher doses of prazosin were required to achieve tumor control in 92.1 or UPMM3 spheroids (Figure 4), suggesting that other factors may play a role in the long-term outcome of prazosin treatment. By using immunofluorescence staining, we confirmed that all cell lines expressed α_1A_- and α_2A_-AR subtypes (Figure 2). However, the expression levels of α-ARs in 3D spheroids may differ from the expression levels in cell lines and may also be influenced by treatment with α-blockers. It remains to be clarified whether, e.g., higher expression levels of α-ARs in the 92.1 and UPMM3 spheroids could have contributed to the long-term results of prazosin treatment. Furthermore, a follow-up study will provide information on the relationship between α-ARs and cytogenetic risk factors such as monosomy 3/BAP1 deficiency based on the histology of UM tumors.

The pronounced responsiveness of MM28, together with the high sensitivity of several primary UM cultures, suggests that α_1_-AR blockade may impair cellular processes important for metastatic colonization or survival. This is consistent with evidence that α_1_-AR antagonists have been reported to disrupt focal adhesion and survival signaling, induce anoikis, and suppress invasion in models of renal cancer [34,35], supporting the broader potential of α_1_-AR modulation to limit metastatic progression.

In addition to the inhibitory effects of prazosin, we observed that phenylephrine, a α_1_-AR agonist widely used in ophthalmology for mydriasis, increased UM growth under certain in vitro conditions (Figure 5). This suggests that α-AR signaling may exert concentration-, cell-, and context-dependent effects. In the context of cancer, the endogenous AR agonists epinephrine and norepinephrine promot progression and metastasis, suggesting a clear link between stress and cancer [36,37]. It is important to note that our in vitro observation does not imply a general clinical cancer risk, as ophthalmic phenylephrine is administered topically at low concentrations and for short durations, with limited penetration into uveal tissues due to tear turnover, corneal and scleral barriers, and rapid clearance [38,39]. In vivo, the ocular microenvironment—including immune surveillance, stromal interactions, and extracellular matrix regulation—provides multiple layers of control that restrain abnormal proliferation, a regulatory context absent in simplified in vitro models.

Still, our study suggests that α_1_-AR signaling may play a role in promoting UM growth, as prazosin and RS13053 were able to prevent phenylephrine-stimulated growth of UM spheroids (Figure 5). Interestingly, α_1_-ARs are coupled to G protein alpha Gq_/11_ signal transduction. However, mutations in either Gq or G_11_ (Table 1, GNAQ, GNA11,) are driving oncogenesis in most UM cases [40]. Our study suggests that even in the context of mutated Gq/_11_, it may be possible to target G protein receptors to counteract oncogenic G protein signaling in UM. In addition, α-AR-independent effects of α_1_-blockers such as prazosin and doxazosin have been described based on their quinazoline structure in non-UM cancer cell models such as renal cell carcinoma or prostate cancer [34,41,42]. The proposed cytotoxic mechanism underlying these α-blockers differed in the various cancers and included VEGF, EGFR/HER2, integrin, TNFα, and other mitochondrial apoptotic-inducing factors [34,41,42]. However, the mechanisms mediating the antitumor activity of α-blockers in UM remain unknown and need to be elucidated in further studies, including animal models such as α_1_-AR-deficient UM mouse models driven by oncogenic GNAQ/GNA11.

Similar to prostate cancer cell lines, in UM spheroids, the relative cytotoxic potency of prazosin and doxazosin was significantly higher compared to terazosin, alfuzosin, silodosin, or tamsulosin [41]. Prazosin showed a cytotoxic effect in a concentration range comparable to that of R-carvedilol, the α_1_-blocking enantiomer of the nonselective β-blocker carvedilol [13]. Of all the agents tested in our studies, however, the α_1A_-AR selective antagonist RS17053 proved to be the most potent as only nM concentrations were needed to block UM spheroid growth. However, RS17053 and R-carvedilol are not clinically available for repurposing in UM therapy. Taken together, our findings position prazosin as the most promising candidate among clinically available α-blockers for repurposing in UM therapy.

To our knowledge, this is the first study addressing α-AR antagonists in UM. However, our results are limited by the selection of a group of cell lines and resulting 3D spheroids, which cannot fully reflect the heterogeneity in patient tumors. To overcome this limitation, we suggest the evaluation of the feasibility of local prazosin treatment of patient tumor samples by using our recently developed xenograft chick embryo model [43,44]. Both the growth of the tumor sample and the tumor cell spread into the liver can be determined in our chick embryo model [43,44]. In further studies, the outcome of prazosin therapy may be evaluated based on tumor growth in small tumors and young patients prior to radiotherapy, taking into account the effects of treatment on vision and the resulting eye discomfort. We propose that prazosin could be a therapy option, particularly when used early in tumor development or as an adjunct to established treatments such as or radiotherapy [12] or tebentafusp [45]. While further in vivo validation is needed, the present work provides new mechanistic and preclinical evidence that targeting α-AR signaling could improve strategies for local tumor control and metastasis prevention.

## 5. Conclusions

Our study has demonstrated that a specific group of α-blockers exhibits antitumor potential for UM. The clinically available α-blockers prazosin and doxazosin, as well as the α-AR antagonist RS17053, were the most effective in eliciting antitumor responses in UM spheroids. These responses included reduced viability, induction of apoptosis, and growth inhibition. In addition, prazosin prevented spheroid outgrowth and long-term survival, as well as spheroid formation. However, in vivo studies, including xenograft chick embryo models or mouse models, are required and planned to determine the effect of α-blockers on tumor tissue samples from patients and healthy tissue. Nevertheless, the results suggest that treatment with α-blockers could be used for local tumor control and metastasis prevention, providing a starting point for new therapeutic approaches in the treatment of uveal melanoma.

## Figures and Tables

**Table 1:** Details of uveal melanoma cell lines.

Cell Line	Origin	Oncogene	Mutations	Chromosome 3	Morphology	Doubling Time	References
92.1	Primary, choroid	GNAQ Q209L	EIF1AX	Disomy-3	Epithelioid	38–58 h	[21,22,23]
Mel270	Primary, cilio-choroid recurrence	GNAQ Q209P	---	Disomy-3, loss 3p24, 3q21.2–3q22	Spindle	43 h	[22,23,24,25]
MM28	Metastatic, liver	GNA11 Q209L	BAP1 deficiency	Loss 3q	Mixed	109 h	[26]
UPMD1	Primary, choroid	GNA11 Q209L	---	Disomy-3	Epithelioid	100 h	[20,22,27]
UPMD2	Primary, cilio-choroid	GNA11 Q209L	---	Disomy-3, partial loss 3q	Epithelioid	150 h	[20,22,27]
UPMM2	Primary, cilio-choroid	GNAQ Q209L	BAP1 deficiency	Monosomy-3	Spindle	150 h	[20,22,27,28]
UPMM3	Primary, choroid	GNAQ Q209P	BAP1 deficiency	Monosomy-3	Mixed	100–150 h	[20,22,27]

## Data Availability

Data are contained within the article.

## Abbreviations

The following abbreviations are used in this manuscript:

AR	Adrenergic Receptor
PE	Phenylephrine
PZ	Prazosin
RS	RS17053
UM	Uveal Melanoma
